# Multimorbidity patterns across race/ethnicity as stratified by age and obesity

**DOI:** 10.1038/s41598-022-13733-w

**Published:** 2022-06-11

**Authors:** Manal Alshakhs, Bianca Jackson, Davina Ikponmwosa, Rebecca Reynolds, Charisse Madlock-Brown

**Affiliations:** 1 Health Outcomes and Policy Program, University of Tennessee Health Science Center, Memphis, TN USA; 2 Health Informatics and Information Management Program, University of Tennessee Health Science Center, 66 North Pauline St. rm 221, Memphis, TN 38163 USA; 3 Center for Health System Improvement, University of Tennessee Health Science Center, Memphis, TN USA

**Keywords:** Diseases, Public health

## Abstract

The objective of our study is to assess differences in prevalence of multimorbidity by race/ethnicity. We applied the FP-growth algorithm on middle-aged and elderly cohorts stratified by race/ethnicity, age, and obesity level. We used 2016–2017 data from the Cerner HealthFacts electronic health record data warehouse. We identified disease combinations that are shared by all races/ethnicities, those shared by some, and those that are unique to one group for each age/obesity level. Our findings demonstrate that even after stratifying by age and obesity, there are differences in multimorbidity prevalence across races/ethnicities. There are multimorbidity combinations distinct to some racial groups—many of which are understudied. Some multimorbidities are shared by some but not all races/ethnicities. African Americans presented with the most distinct multimorbidities at an earlier age. The identification of prevalent multimorbidity combinations amongst subpopulations provides information specific to their unique clinical needs.

## Introduction

Multimorbidities become more prevalent as individuals age, and they have been associated with substantial burden and increased mortality^1^. The treatment of patients with multimorbidity can be complicated by the involvement of multiple medical specialties^2^. These patients are also at risk of repeated hospitalization, polypharmacy, adverse drug events, and increased care dependence^3^. Obesity exacerbates multimorbidity prevalence and further contributes to increased healthcare utilization and adverse health outcomes. Internationally, the medical community has called for a greater understanding of multimorbidity patterns^4^. Identifying homogeneous multimorbidity subgroups has been theorized as a way to develop and target interventions more effectively^5^. Studies have also shown that specific racial and ethnic groups are at greater risk of poor health outcomes^6^.

To minimize the adverse effects of multimorbidity, patients need proactive, precise, and patient-centered care plans that explicitly address patients' most critical needs with the most prevalent multimorbidity combinations^7^. Further, providers should be aware of the most likely multimorbidity combinations to develop and coordinate appropriate care plans when treating patients with these disease groupings^8^. It is crucial to identify specific multimorbidity patterns because the impact of multimorbidity on health-related quality of life varies for different combinations^9^.

Although previous studies have identified factors that may impact multimorbidity, most relevant studies were not conducted in the US, and few studies account for obesity or age^2,10^. Additionally, there remains a need to identify homogeneous combinations among race/ethnicities. Most previous research identified multimorbidity patterns using counts or cluster analysis and are disease specific^11^. These approaches are limited as there exists "tremendous diagnostic heterogeneity, variation in the number of chronic conditions, and the severity of illness characterized the multiple chronic conditions population making identification of multimorbidity trends difficult"^12^. One research team using clustering for multimorbidity pattern identification state "though recognition of general patterns of disease co-occurrence is useful for policy planning, the heterogeneity of persons with significant multimorbidity (≥ 3 conditions) defies neat classification. A simple count of conditions may be preferable for predicting usage"^13^. Chong et al. say the benefits of segmenting patients by multimorbidity patterns will be the "facilitation of healthcare service planning, promotion of the evaluation of health service innovations, and improving care integration"^14^. Understanding which diseases cluster together most frequently will lead to the understanding of which disease clusters have the "most significant impact on essential patient outcomes"^15^. Though it may be tempting to assume patient groups need care plans tailored to each disease, research has shown that fragmented care can lead to ineffective and potentially harmful interventions^16^.

Several studies have identified the prevalence of specific multimorbidity patterns for clusters of two or more diseases. Our previous work identified the most prevalent multimorbidity patterns of two or more diseases for a cohort with obesity^17^. Held et al. used association rule analysis mining to find dyads and triads of diseases for a cohort of men of older age in Australia^18^. Van den Bussche et al. identified combinations of 3 multimorbidities in a sample of German elderly patients^19^. While these studies shed considerable light on how diseases tend to combine, they are limited in that they include only special populations and are not stratified by factors that are likely to impact the pattern of multimorbidity.

Disparities in multimorbidity exist by race and ethnicity. Recent studies have found that African Americans tend to have higher rates of multimorbidity compared with Caucasians, while rates are higher among Caucasians compared with Asians^6^. Race is also associated with fasters rates of multimorbidity development. One study demonstrated that African Americans developed multimorbidity about four years earlier than Caucasians in a middle-aged adult population^6^. An analysis of multimorbidity networks similarly found that African Americans have the most densely connected network at the organ-level, followed by Caucasians and Native Americans^20^. There is an urgent need to understand the specific prevalence of multimorbidities between races/ethnicities. These gaps motivate the need for similar research to be conducted within a US patient population, and further emphasize the need to evaluate the effect of race/ethnicity on multimorbidity patterns.

Researchers have also demonstrated that multimorbidity can vary by sociodemographic factors. In a Canadian study among middle-aged adults, the prevalence of multimorbidity increased from 29.7% in individuals that were 45–49 years of age to 52% for the group 60–64^2^. Differences in multimorbidity patterns among men and women have also been observed in a Spanish population of adults over 65 years of age^2,21^. Further, a cross-sectional study conducted in Singapore reported an association between multimorbidity and increasing age, lower socio-economic status, female sex, and mental disorders^10^.

Although many associations with sociodemographic characteristics and multimorbidity have been found, among the most significant are age and obesity^22,23^. Increasing obesity rates contribute to the growing prevalence of multimorbidity worldwide. The prevalence of multimorbidity in persons with obesity exceeds 60% in the US^17,24^. Compared with patients of normal weight, patients with obesity have an increased risk of developing multimorbidity^1,25^. Multimorbidity combinations containing obesity, specifically, may result in increased social isolation and vulnerability^26^, poorer outcomes, increased hospitalization and healthcare costs, compared with other multimorbidity combinations^2,27^. Interestingly, more middle-aged adults than older adults tend to be afflicted with obesity and multimorbidity. They tend to live longer with multimorbidity and associated complications, warranting special attention to obesity-associated multimorbidity within this specific population^25^.

Our work will address different multimorbidity patterns across race/ethnicity in the United States. Although we are not the first to use a frequent item-set algorithm to assess multimorbidity^18^, our study is the first to classify distinct prevalent multimorbidities by race/ethnicity and stratified for obesity and age. Disadvantaged populations are disproportionately affected by multiple chronic conditions^28^, which may affect their multimorbidity patterns. Therefore, we aim to identify patterns of morbidity that may be prevalent for only some groups.

The objectives of the research are to:

- Identify prevalent multimorbidity patterns by race/ethnicity category within middle-aged and elderly cohorts which will be further subdivided into with and without obesity groups.
- Compare individual disease prevalence of multimorbidities found in all race/ethnicity.
- Identify multimorbidities found in more than one but not all race/ethnicity.
- Identify multimorbidities that are distinct to one race/ethnicity.
- Assess potential multimorbidity disease burden across cohorts.

## Methods

### Design

This cross-sectional study employed data collected in 2016–2017 and stored the Cerner HealthFacts data warehouse, which includes patient encounter records for over 70 million patients treated at hospitals and clinics throughout the United States between 2001 and 2017. This dataset includes medical histories, diagnoses, laboratory information, prescriptions, patient demographics, clinic type, procedures, and surgical data documenting over 490 million encounters from 792 non-affiliated health care systems. It includes outpatient, inpatient, emergency, and other encounter types. The inclusion criteria for patients were: (1) Age 45+, (2) at least one clinical encounter during 2016–2017 with Body Mass Index (BMI) values between 18.5 and 206 present, (3) assigned race/ethnicity, (4) ≥ 1 International Classification of Diseases, 10th revision, Clinical Modification (ICD-10-CM) diagnosis code that indicates a medical condition, and (5) having no ICD-9-CM classification documented during this timeframe. We chose a two-year period for the study of multimorbidity to increase the likelihood that each patient had each morbidity concurrently. For each patient, diagnoses were aggregated across all visits for this period. We did not require patients to have more than one encounter as research shows healthcare use and access vary by race^29^, and being too restrictive could limit the representativeness of our sample.

### Ethical considerations

The data are de-identified and exclude the 16 identifiable variables that necessitate Institutional Review Board (IRB) approval for access. Because of the de-identified nature of the data, this study is not considered human subject research. The University of Tennessee Health Science Center IRB determined that this research qualifies for 'Not Human Subjects Research' status according to the policies of the National Institutes of Health Office of Human Subjects Research. This research was performed in accordance with all relevant guidelines/regulations.

### Variables

Our outcome variables were multimorbidity patterns prevalent within each racial/ethnic category stratified by age and obesity status. There is no international consensus defining multimorbidity^30^. We defined multimorbidity as the presence of two or more chronic conditions within one individual^31^. For different definitions, the duration a patient must have a chronic condition varies from 3 months to a year (or may not list a specific duration)^32^. Datasets derived from EHRs do not typically capture the duration that a patient may suffer from a diagnosis which may extend until the end of life. Several different organizations maintain lists of chronic conditions that are used in research, but each one omits some chronic conditions^33^. Our aim is to include a broad array of conditions to better reflect patient disease states by using all ICD-10-CM codes representing conditions that can be chronic. The ICD-10-CM codes we considered include those from the following clinical categories: E00–E89, endocrine, nutritional, and metabolic diseases; F01–F99, mental, behavioral, and neurodevelopmental disorders; G00–G99, diseases of the nervous system; I00–I99, diseases of the circulatory system; J00–J99, diseases of the respiratory system, K00–K95, diseases of the digestive system; M00–M99, diseases of the musculoskeletal system and connective tissue; and N00–N99, diseases of the genitourinary system. The specific subcodes we considered are described in the text and enumerated in Supplementary Table 2. Patients were considered positive for individual diseases or comorbidities if their record included one or more ICD-10-CM diagnostic code(s) within the respective broad disease category during 2016–2017. For example, a patient with ICD-10-CM codes E11 and I10 would be positive for type II diabetes and essential (primary) hypertension, respectively. All sub-classifications of diseases were included under the umbrella of their broad disease code. For example, code E11.0 (type 2 diabetes mellitus with hyperosmolarity) would be categorized under the broader parental code E11 (type 2 diabetes mellitus).

Our analysis includes multimorbidities based on prevalent codes as prevalence-based selection of ICD-10-CM codes in multimorbidity research has been shown to be robust^34^. Patients were classified with obesity if they had an average BMI of 30+ during the study period, and without obesity if their BMI was less than this cutoff. Patients were further stratified into middle-aged if they were between 45 and 64, and elderly if they were 65+. We considered a BMI value to be valid if it was less ≤ 206 as the highest recorded BMI values are in the low 200 s between 206 and 224^35,36^. We recognize that due to the size of our sample, outliers are unlikely to have a noticeable impact on our results. Since BMI was used to stratify our results, we excluded it as an outcome variable.

Racial categories were based on those present within the Cerner HealthFacts data warehouse. Because we were unable to separate patients listed as Asian/Pacific Islander into either Asian or Pacific Islander categories, we decided to merge Asian, Pacific Islander, and Asian/Pacific Islander categories into one category. To ensure that we could identify the groups from which any category was drawn, we excluded patients from the study if their race/ethnicity was missing or listed as other. One category, Mid-Eastern Indian, was removed as the authors were unclear as to its meaning. The final racial categories used in the analysis were African American, Caucasian, Asian/Pacific Islander, Biracial, Hispanic, and Native American.

### Identifying multimorbidity patterns

We used a Spark distributed cluster for our analysis. We used frequent itemset detection to find combinations of diseases above the threshold of 5% prevalence. This algorithm identified groups of 2 + diagnoses that appear together in the dataset for at least 5% of patients within each race/ethnicity age obesity-level cohort. Our rationale for setting support to 5% is as follows: the tremendous diagnostic heterogeneity and variation in the number of chronic conditions for the multimorbidity population suggests there will be many combinations of low frequency. Therefore, we did not want to set support too high. Frequent itemset detection can reveal disease clusters in which all patients share all group attributes. Below is an example problem where the minimum support (threshold of prevalence) is 60%.

Patient lists of ICD-10-CM diagnosis codes where each row represents one patient:

I10, G47, E78

I10, E78

G47

I10, R06

I10, R06, E78

The frequent patterns whose frequencies are above the 60% threshold of minimum support are:

I10

E78

I10, E78

Finding frequent itemsets can be computationally expensive on large datasets. However, the parallel frequent pattern growth (FP‐growth) algorithm is an efficient distributed frequent itemset mining algorithm that reduces the number of candidate itemsets when used on a distributed cluster to find patterns on large datasets^37^.

The FP‐growth algorithm is a scalable frequent itemset algorithm frequently used on large datasets^38^. The parameters for the FP‐growth algorithm are as follows: Let I = {P1, P2…Pm} represent a set of m diagnoses. Each patient's diagnoses list L for the study period contains a set of diagnoses such that L ⊆ I. The support (occurrence frequency) of a pattern A, where A is a set of diagnoses is the number of patient lists containing A. A pattern is considered frequent if A's support is greater than or equal to a pre‐defined minimum support threshold, ξ. We used the SparkR implementation of this algorithm^39^. We applied the FP-growth algorithm on each cohort stratified by race/ethnicity, age, and obesity level. We compared results across races/ethnicities for each age range by obesity level. We compared the disease combinations shared by all races/ethnicities, those shared by some, and those unique to one group for each age/obesity level.

We calculated confidence intervals for multimorbidity prevalence for diseases shared across races/ethnicities to identify which races/ethnicities may have similar prevalence. We used the Clopper–Pearson method to generate binomial proportion confidence intervals^40^. To compare prevalence rates by race, we used the g‐test of independence. These statistical analyses were performed using the R DescTools package^41^. Sensitivity analysis included using the Kruskal–Wallis ANOVA for medians of BMI across race for each age/weight class to determine if our findings could be impacted by one race having a higher median BMI within the weight class, which could lead to skewed results. We used the WRS2 R package for this test^42^. The g-test were used to determine statistical significance. P values < 0.05 were considered to be statistically significant.

## Results

### Sample: middle-aged and elderly cohorts stratified by race/ethnicity

A total of 1,212,956 patients matched our criteria in the middle-aged cohort and 1,003,498 patients in the elderly cohort. Supplementary Table 1 shows the patient population for this study. Our patient population's average number of visits was seven, and 76% of patients had 2 + encounters. In each cohort, most patients were Caucasian (77% and 87% respectively), followed by African American (19% and 10% respectively), as shown in Table 1. Patients listed as Asian/Pacific Islander accounted for 2% of the Middle-aged cohort and 2% of the Elderly cohort. Native Americans accounts for 1% in the middle-aged cohort, and < 1% in the elderly cohort. Both the Biracial and Hispanic cohorts account for < 1% of the samples in both age groups. Except for Asians/Pacific Islanders, most middle-aged patients for each race/ethnicity were in the with obesity category. For the elderly cohorts, there were more patients with obesity for all three categories.

**Table 1:** Demographic breakdown across age and weight class.

Race/ethnicity	Middle aged			Elderly		
	Total % of population (total population)	Without obesity	With obesity	Total % of population (total population)	Without obesity	With obesity
African American	19% (235,612)	42% (98,759)	58% (136,859)	10% (102,138)	55% (55,745)	45% (46,393)
Asian/Pacific Islander	2% (21,013)	76% (15,947)	24% (5066)	2% (15,427)	84% (12,982)	16% (2445)
Biracial	< 1% (1220)	49% (596)	51% (624)	< 1% (506)	57% (288)	43% (218)
Caucasian	77% (936,472)	48% (448,299)	52% (488,173)	87% (876,538)	59% (520,821)	41% (355,717)
Hispanic	< 1% (7008)	46% (3200)	54% (3808)	< 1% (3227)	59% (1895)	41% (1332)
Native American	1% (11,578)	38% (4443)	62% (7135)	< 1% (5662)	52% (2963)	43% (2699)

Table 2 shows the number of total multimorbidities at the 5% threshold for prevalence across weight class for each race/ethnicity cohort with the number of distinct and overall multimorbidities for each by age and weight cohort. We found African Americans patients have the highest number of total multimorbidities and the most distinct multimorbidities for each age/weight group. Middle-aged without obesity cohorts have the lowest number of multimorbidities for each race/ethnicity, and Elderly with obesity have the most. Supplementary Table 2 describes the diagnosis codes and corresponding diagnoses included in the observed multimorbidity patterns.

**Table 2:** The number of multimorbidity patterns by race/ethnicity at 5% threshold across weight class.

Race/Ethnicity	Middle aged Without obesity		Middle aged With obesity		Elderly Without obesity		Elderly With obesity	
	Overall	Distinct	Overall	Distinct	Overall	Distinct	Overall	Distinct
African American	17	6	50	20	112	45	157	37
Asian/Pacific Islander	4	0	15	0	41	1	98	2
Biracial	2	0	11	0	58	3	65	1
Caucasian	5	0	24	2	58	8	95	9
Hispanic	6	0	12	0	44	0	63	0
Native American	13	1	22	3	60	4	107	3

### Multimorbidities shared across all races/ethnicities

**Figure 1:**
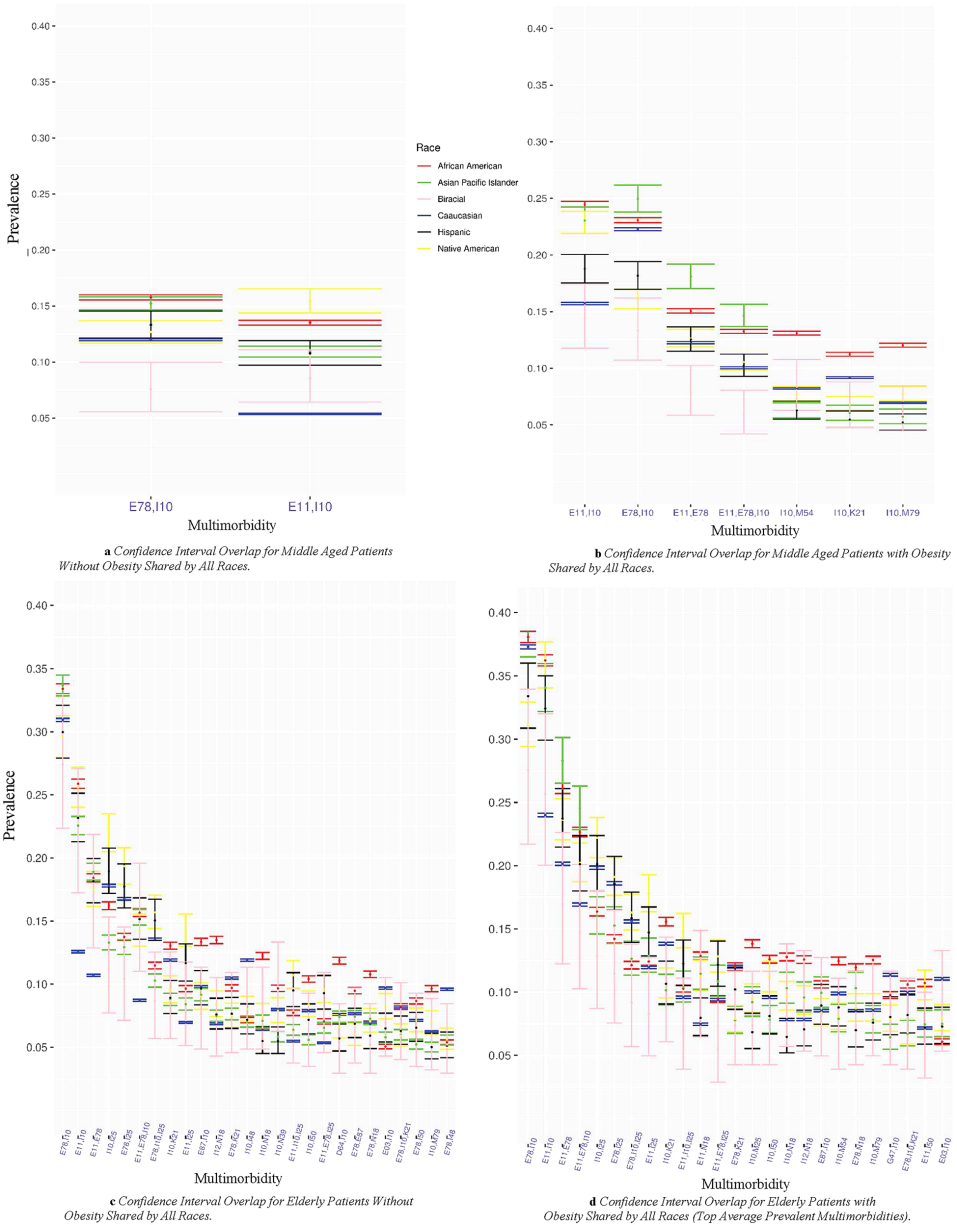
(**a**,**b**) Demonstrate the 95% confidence interval overlap for shared multimorbidities among middle aged patients without obesity and patients with obesity, respectively, at 5% prevalence threshold. The g-test of independence was calculated resulting in all significant results with p < 0.05. (**c**,**d**) Demonstrate the 95% confidence interval overlap for shared multimorbidities among elderly patients without obesity and patients with obesity, respectively, at 5% prevalence threshold. The chi square test was calculated resulting in all significant results with p < .05 except for the multimorbidity patterns circled in red. Disease Clinical Categories; E: Endocrine, nutritional and metabolic, F: Mental, behavior and neurodevelopmental, G: Nervous System, I: Circulatory system, J: Respiratory system, K: Digestive system, N: Genitourinary system, M: musculoskeletal system and connective tissue. (**d**) Demonstrates shared multimorbidities with average prevalence ≥ 0.08.

Figure 1a–d show the confidence intervals for multimorbidity patterns shared across all race/ethnic groups analyzed within each age/obesity cohort. Gray sectors indicate the combination was not prevalent above the 5% threshold for a give group. The multimorbidity patterns shared across all racial and ethnic groups included one or more of the following ICD-10-CM codes with the corresponding diagnoses: I10: Hypertension, E78: Lipidemia, or E11: Diabetes. As described in Supplementary Table 2. There were only two shared patterns across all races/ethnicities in the middle-aged cohort without obesity (Fig. 1a). Caucasians had the lowest prevalence for the E11: Diabetes, I10: Hypertension combination, and Biracial had the lowest prevalence for the E78: Lipidemia, I10: Hypertension combination. In the middle-aged patients with obesity cohort, there were six multimorbidity patterns. In this cohort, the E78: Lipidemia + I10: Hypertension and E11: Diabetes + I10: Hypertension combinations had the highest prevalence for each race/ethnicity. There were only two clinical categories (E: Endocrine, nutritional and metabolic, and I: Circulatory system) represented in the middle-aged cohort without obesity, while that increased to 7 in patients with obesity (including K: Digestive system and M: Musculoskeletal system and connective tissue).

For five of the multimorbidity patterns observed for middle-aged patients with obesity (Fig. 1b), the 95% confidence interval for the African American patients did not overlap with any other racial/ethnic group with one exception, E11: Diabetes + I10: Hypertension. For the E78: Lipidemia and E11: Diabetes diagnosis codes, the 95% confidence interval overlaps with that of the Asian/Pacific Islander cohort. For patients without obesity, there is an increase from two patterns in the middle-aged to 26 patterns in the elderly.

Results for the elderly cohorts appear in Fig. 1c,d and Supplementary Fig. 1. There are 26 patterns for the without obesity cohort and 37 for the with obesity cohort. Due to the difficulty in interpreting so many patterns, we have limited the set that appear in Fig. 1d to only those with prevalence equal to or above 0.08%. In the without obesity cohort (Fig. 1c), five patterns include I25: Heart Disease, and two include N18: Chronic kidney disease (CKD). For the E78: Lipidemia + I10: combination, the Asian/Pacific Islander and African American cohort have the highest estimates. For three patterns, the Caucasian cohort estimate error bars do not overlap with any other group and are the lowest.

In the elderly patients with obesity cohort (Fig. 1d and Supplementary Fig. 1), 25 multimorbidity patterns were present. The following diagnoses appeared in 3 or more multimorbidity patterns: I10: Hypertension (19 patterns), E78: Lipidemia (13patterns), E11: Diabetes (12 patterns), I25: Heart disease (8 patterns), N18: Chronic kidney disease (6 patterns), K21: GERD (4 patterns), I50: Heart failure (3 patterns), and E87: Other disorders of fluid, electrolyte and acid–base balance (3 patterns). The elderly cohort patterns include three new clinical categories, D: Disease of blood and blood-forming organs, N: Diseases of the genitourinary system, and G: Diseases of the nervous system.

### Multimorbidities shared among some races/ethnicities

**Figure 2:**
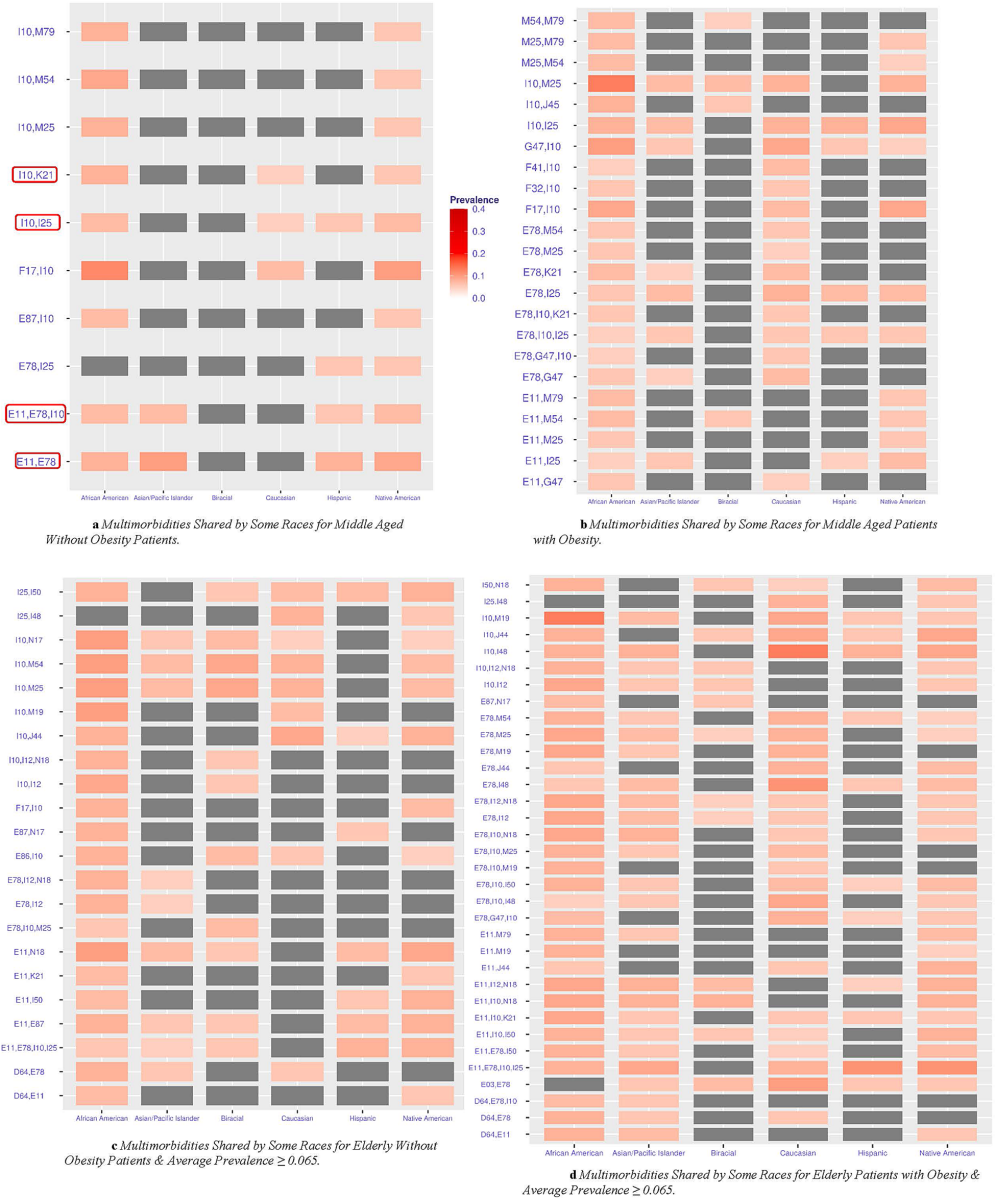
(**a**,**b**) Show shared multimorbidities across two or more races in middle aged patients without and with obesity, respectively, at 5% prevalence threshold. Gray sectors indicate the combination was not prevalent above the 5% threshold for a given group. The g-test of independence was calculated resulting in all significant results with p < 0.05 except for red boxed multimorbidity pattern(s) indicating non-significant result. (**c**,**d**) Demonstrate the shared multimorbidities among two or more races in the elderly patients without and with obesity, respectively, at 5% prevalence threshold. The g-test was calculated resulting in all significant results with p < 0.05 except for the multimorbidity patterns circled in red. Disease Clinical Categories; E: Endocrine, nutritional and metabolic, F: Mental, behavior and neurodevelopmental, G: Nervous System, I: Circulatory system, J: Respiratory system, K: Digestive system, N: Genitourinary system, M: musculoskeletal system and connective tissue.

Multimorbidities shared across two or more races/ethnicities, but not all, in the middle-aged and elderly cohorts are shown in Fig. 2a–d. Gray sectors indicate the combination was not prevalent above the 5% threshold for a given group. A total of 10 multimorbidity patterns for patients without obesity and 23 for patients with obesity appeared in the middle-aged patient cohort (Fig. 2a,b). Clinical categories not represented in the middle-aged cohort shared by all but appear in the set of those shared by some are F: Mental, Behavioral and Neurodevelopmental disorders and J: Diseases of the respiratory system. All combinations for the middle-aged included at least one of the following diseases: E11: Diabetes, I10: Hypertension, E78: Lipidemia, M54: Dorsalgia, or M25: other joint disorders. In the middle-aged cohort without obesity (Fig. 2a), African Americans and Native Americans shared all multimorbidity patterns except for the E78: Lipidemia I25: Heart disease combination. African Americans and Native Americans had higher prevenances of the F17: Nicotine dependence-I10: Hypertension combination than the Caucasian cohorts in both the with and without obesity middle-aged samples (Fig. 2a–d). In the middle-age patients with obesity cohort (Fig. 2b), the multimorbidities with the highest prevalence for each group include I10: Hypertension, I25: Chronic ischemic heart disease, G47: Sleep disorders, and K21: Gastro-esophageal reflux disease appeared in several combinations. Patterns including musculoskeletal system diseases and connective tissue (those that start with M) were prevalent for the African American, Native American, Asian/Pacific Islander, and Biracial cohorts only. Middle-aged with obesity African Americans and Native American groups (Fig. 2b) have a similar prevalence for the following combinations: E11: Diabetes + M25: Other joint disorders; E11: Diabetes + M79: Other and unspecified soft tissue disorders; M25: Other joint disorders + M79: Other and unspecified soft tissue disorders, and M25: Other joint disorders + M54: Dorsalgia (Fig. 2b). The only two combinations present for the Asian/Pacific Islander without obesity (Fig. 2a) include both diabetes E11: Diabetes and E78: Lipidemia.

Results for the elderly cohorts appear in Fig. 2c–d and Supplementary Figs. 2, 3. A total of 47 multimorbidity patterns for patients without obesity and 90 for patients with obesity appeared in the elderly patient cohort. Due to the difficulty in interpreting so many patterns, we have limited the set that appear in Fig. 2c–d to only those with prevalence equal to or above 0.065. The rest of the patterns for the without obesity cohort appear in Supplementary Fig. 2, and those for the with obesity appear in Supplementary Fig. 3. No new clinical categories are represented. Combinations in the cohort without obesity (Fig. 2c and Supplementary Fig. 2) include diagnoses not found in the middle-aged cohort, including N17: Acute Kidney Failure and F17: Nicotine dependence. In the elderly cohort without obesity (Fig. 2c and Supplementary Fig. 2), there are 11 patterns comprised of three morbidities and eight comprised of four morbidities. The patients in the elderly with obesity cohort (Fig. 2d and Supplementary Fig. 3) exhibited 46 multimorbidity combinations comprised of three multimorbidities and five comprised of four multimorbidities. In the cohort without obesity, the three most prevalent patterns comprised three diagnoses (Fig. 2c) all include one to two diagnoses in the I: Diseases of the circulatory system category. These patterns are not prevalent for the Caucasian cohort**.** The highest prevalent pattern with four diagnoses (Fig. 4c) includes I25: Heart Disease and is prevalent for all groups except Caucasians. In the elderly cohort, the same highest prevalent pattern of four morbidities, E11: Diabetes + E78: Lipidemia + I10: Hypertension + I25: Heart Disease, is prevalent for all groups except Biracial. In the cohort without obesity, all patterns of three or four are prevalent for the African American cohort except for E03: Other Hypothyroidism + E78: Lipidemia + I10: Hypertension (Supplementary Fig. 2). That combination is prevalent for the Caucasian and Native American cohorts only. Five of the top prevalent patterns of three morbidities in the elderly cohort with obesity include N18: Chronic Kidney Disease. The combination of I10: Hypertension + J44: Other chronic obstructive pulmonary disease (COPD) was prevalent for all groups except Asian/Pacific Islander and Biracial in the without obesity patients (Fig. 2c) and for all except Asian/Pacific Islander in the elderly with obesity patients (Fig. 2d). F17: Nicotine dependence appeared in the African American and Native American elderly cohort without obesity (Fig. 2c), yet it did not appear in any shared pattern in elderly patients with obesity (Fig. 2d).

### Multimorbidities distinct to one race/ethnicity

**Figure 3:**
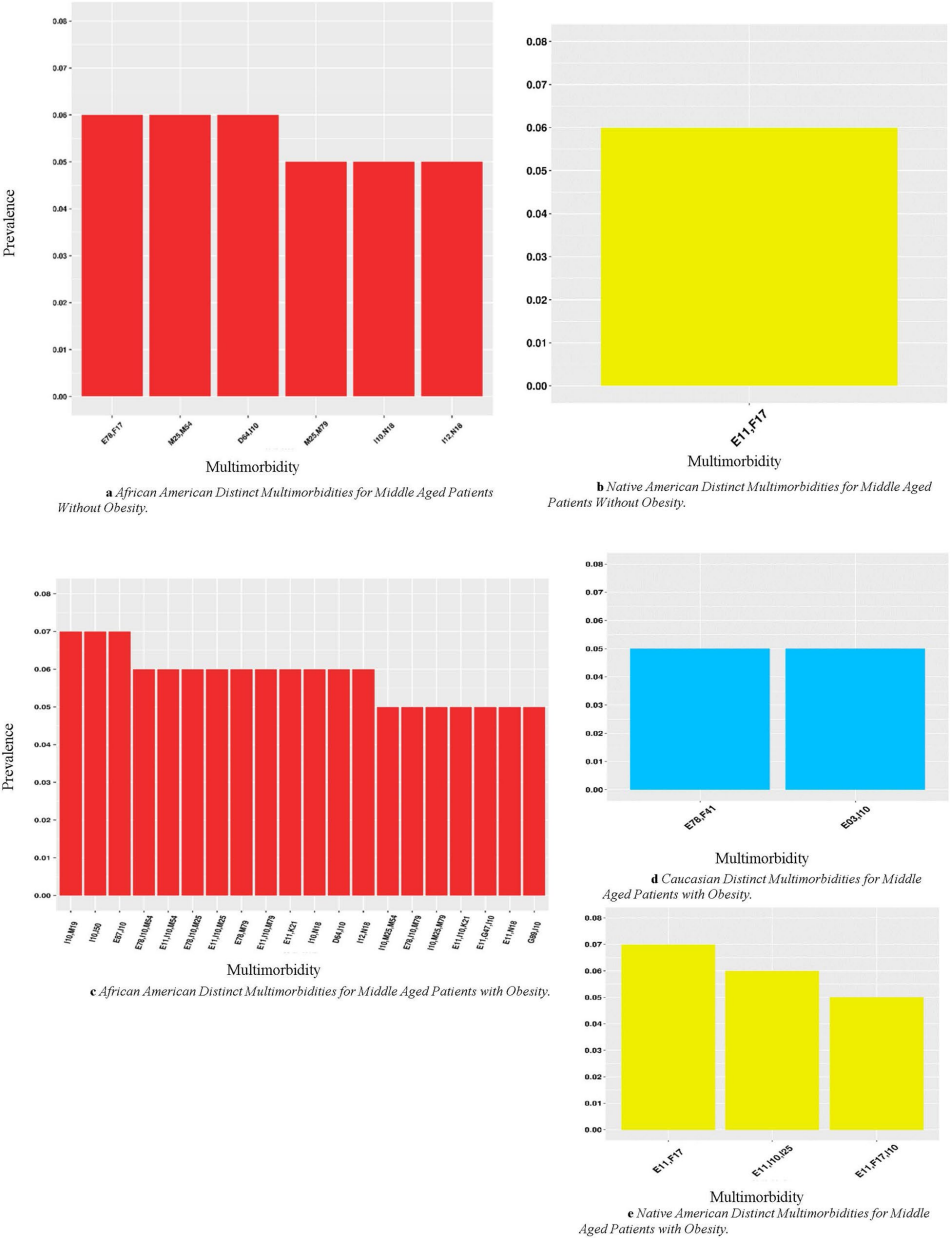
(**a**,**b**) Demonstrates distinct multimorbidities across races in middle-aged without obesity at 5% prevalence threshold. Gray sectors indicate the combination was not prevalent above the 5% threshold for a given group. (**c–e**) Demonstrate the distinct multimorbidities for each race in middle aged patients at 5% prevalence threshold. Disease Clinical Categories; D: Blood and immune mechanism disorders, E: Endocrine, nutritional and metabolic, F: Mental, behavior and neurodevelopmental, G: Nervous System, I: Circulatory system, J: Respiratory system, K: Digestive system, N: Genitourinary system, M: musculoskeletal system and connective tissue. The g-test of independence was calculated resulting in all significant results with p < 0.05.

We also identified distinct multimorbidities unique to a specific racial/ethnic group in both middle-aged and elderly cohorts (Fig. 3a–d). Gray sectors indicate the combination was not prevalent above the 5% threshold. The African American cohort had the most distinct multimorbidity patterns in both weight groups in the middle-aged cohort (Fig. 3a,c). They also had distinct multimorbidity patterns appear with F17: Nicotine dependence or N18: Chronic kidney disease (CKD) in both cohorts. In the middle-aged patients with obesity cohort (Fig. 3c), African Americans had K21: GERD, D64: Other anemias, I50: Heart failure, and musculoskeletal disorders (those starting with M) appearing in several distinct multimorbidity patterns. The cohort without obesity had ten distinct patterns of 3 morbidities. The Native American middle-aged cohort (Fig. 3b,e) had F17: Nicotine dependence appear in a distinct multimorbidity pattern in both cohorts. Each pattern for the Native American includes E11: Diabetes in both with and without obesity groups. Additionally, in the cohorts with obesity, Native Americans had two distinct patterns of three diagnoses (Fig. 3e). For the middle-aged cohort, the Caucasian group had two distinct patterns among the patients with obesity (Fig. 3d). Both include a diagnosis in the E: Endocrine, nutritional and metabolic. One includes F41: Other anxiety disorders.

**Figure 4:**
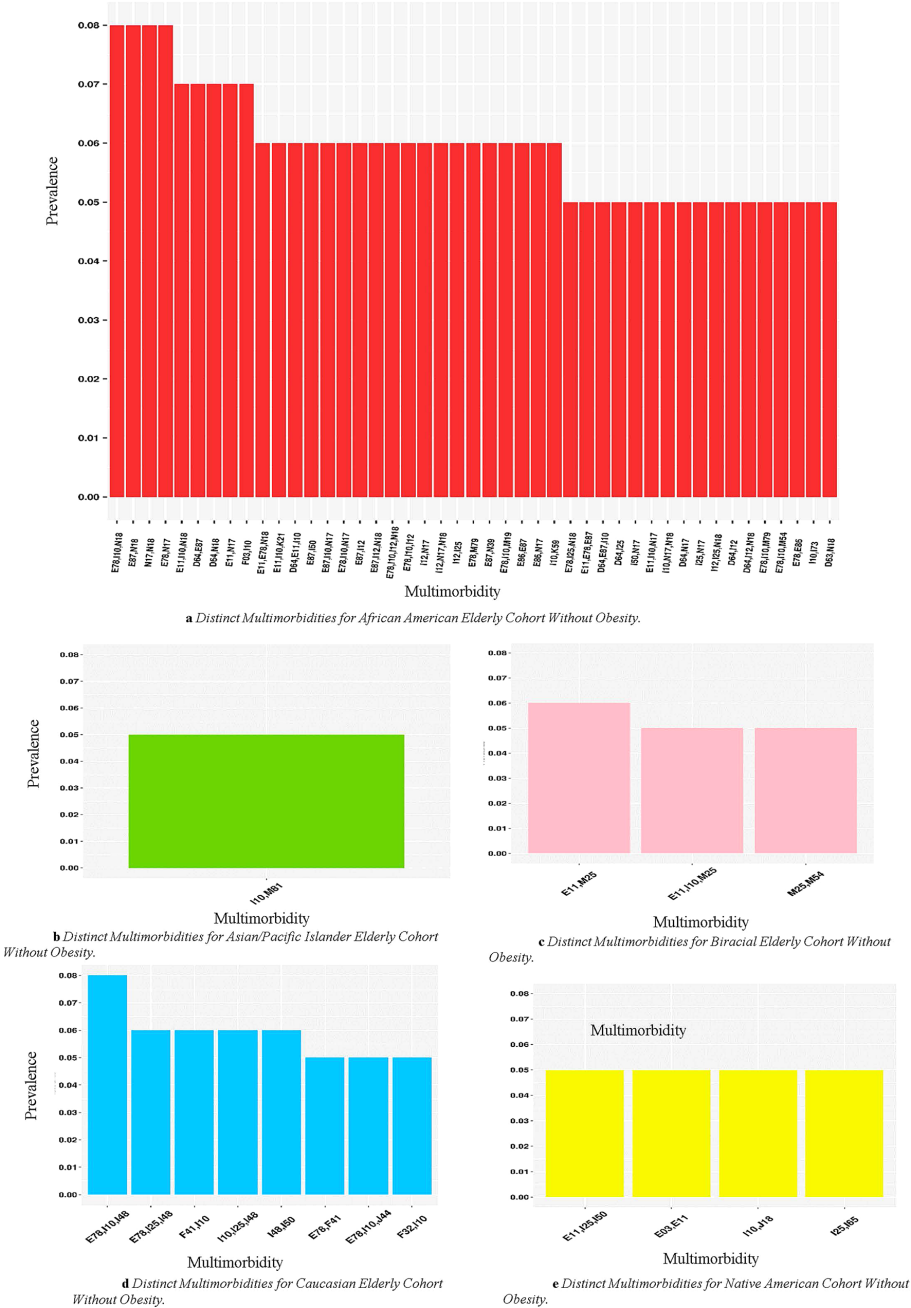
(**a**–**e**) Demonstrate distinct multimorbidities across races in elderly patients without obesity at 5% prevalence threshold. Disease Clinical Categories; D: Blood and immune mechanism disorders, E: Endocrine, nutritional and metabolic, F: Mental, behavior and neurodevelopmental, G: Nervous System, I: Circulatory system, J: Respiratory system, K: Digestive system, N: Genitourinary system, M: musculoskeletal system and connective tissue. The g-test of independence was calculated resulting in all significant results with p < 0.05.

**Figure 5:**
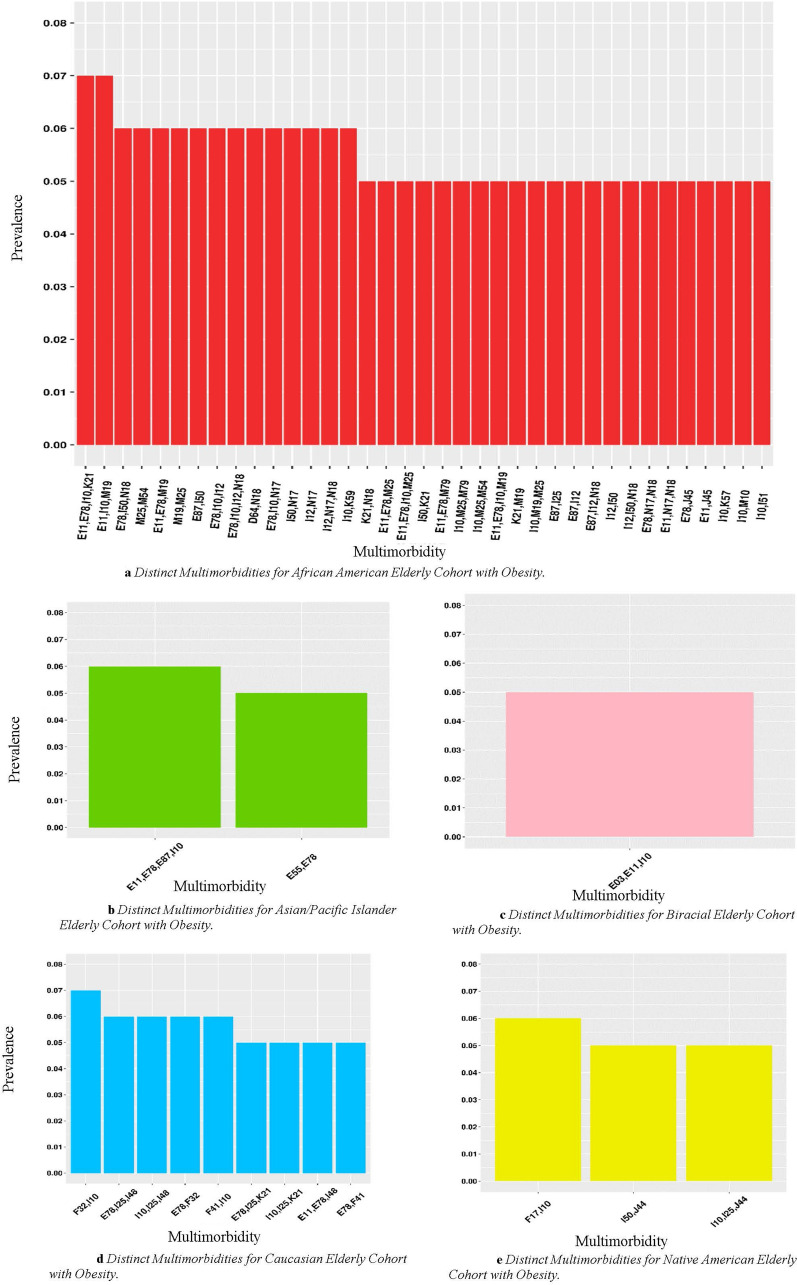
(**a**–**e**) Demonstrate distinct multimorbidities across races in elderly patients with obesity at 5% prevalence threshold. Disease Clinical Categories; D: Blood and immune mechanism disorders, E: Endocrine, nutritional and metabolic, F: Mental, behavior and neurodevelopmental, G: Nervous System, I: Circulatory system, J: Respiratory system, K: Digestive system, N: Genitourinary system, M: musculoskeletal system and connective tissue. The g-test of independence was calculated resulting in all significant results with p < 0.05.

In the elderly, the African American cohort without obesity (Fig. 3c) had many combinations that combined with I10: Hypertension, E78: Lipidemia, or E11: Diabetes, including N18: Chronic kidney disease (CKD), I12: Hypertensive chronic kidney disease, E87: Other disorders of fluid, electrolyte and acid–base balance, F17: Nicotine dependence, K21: GERD, D64: Other anemias, and I50: Heart failure. In the cohort without obesity (Fig. 4a), there are 21 multimorbidities comprised of 3 or 4 diseases. In the cohort without obesity (Fig. 4a), there are 21 multimorbidities comprised of three or four diseases and 19 in the cohort with obesity (5a). Caucasians had F41: Anxiety Disorders and F32: Depressive episode appearing in distinct multimorbidity patterns in the elderly cohorts with and without obesity (Figs. 4d, 5d). This group also had I48: Atrial fibrillation and flutter appear in distinct multimorbidity patterns in the elderly cohorts (Figs. 4d, 5d). In the cohort without obesity (Fig. 4d), there are four multimorbidities comprised of three or four diseases as five in the cohort with obesity (Fig. 5a). For the Asian/Pacific Islander, without obesity group (Fig. 4b), the single pattern displayed includes I10: Hypertension and M81: Osteoporosis without current pathological fracture. For the cohort with obesity, the Asian/Pacific Islander cohort (Fig. 5b) had one pattern of size four. The two patterns for this cohort are composed of I10: Hypertension and diseases in the E: Endocrine, nutritional, and metabolic clinical category. For the Biracial without obesity cohort (Fig. 4c), all patterns include musculoskeletal disorders (those starting with M). The only distinct pattern for the Biracial cohort with obesity (Fig. 5c) includes E03: Other hypothyroidism. In the Native American without obesity cohort (Fig. 4e), the combinations include I50: Heart Failure, J18: Pneumonia, and I65: Occlusion and stenosis of precerebral arteries, not resulting in cerebral infarction. In the cohort with obesity (Fig. 5c), patterns include F17: Nicotine dependence and J44: Other chronic obstructive pulmonary disease (COPD). For both the Biracial and Native American samples in the cohort without obesity (Figs. 4c, e), there is one pattern of three morbidities and one in the cohort with obesity (Fig. 5c, e).

To determine whether any one racial/ethnic group might have an excessively high or low median BMI within a given weight class, we performed Kruskal–Wallis ANOVA to compare median BMI values across race/ethnicity for each age-group and weight class. Median values by race/ethnicity appear in Table 3. The results of the ANOVA analysis showed that the difference in median BMI values for each age or weight class was statistically significant (p < 0.05). For the cohorts without obesity, the difference between each racial/ethnic group was just one point (Table 3). All patient cohorts without obesity exhibited median BMI values of 25–26, which fall into the overweight weight class. For middle-aged patients with obesity, the median BMI value for each racial group was 33–36 (Table 3). For the elderly cohort without obesity, all median values are between 24 and 25. For the elderly with obesity cohort, all medians are between 32 and 34 (Table 3).

**Table 3:** Median BMI across race/ethnicity for each weight class.

Race/Ethnicity	Middle aged Without obesity	Middle aged With obesity	Elderly Without obesity	Elderly With obesity
	Median	Median	Median	Median
African American	25	36	25	34
Asian/Pacific Islander	25	33	24	32
Biracial	26	35	25	33
Caucasian	26	35	25	34
Hispanic	26	34	25	33
Native American	26	35	25	34

## Discussion

The present study identified the most prevalent multimorbidity patterns across races/ethnicities, stratified by age and obesity, and compared individual disease prevalence across age cohorts. It also assessed multimorbidity disease prevalence across cohorts. Our findings demonstrate that even after stratifying by age and obesity, there are differences in multimorbidity prevalence across races/ethnicities, and some combinations are distinct to race/ethnic groups. Although we are not the first to use a frequent itemset algorithm to assess multimorbidity^18^, our study adds to the current body of knowledge by examining the prevalence of *specific* multimorbidity patterns by racial/ethnic category, stratifying by age and obesity status. Many of the identified disease combinations have likely not been studied, as identifying unique patterns among races/ethnicities is unprecedented. Our study showed that the common morbidities present in disease combinations across all races/ethnicities were lipidemia, hypertension, and diabetes regardless of age or obesity level. Multimorbidity increased with age in both with and without obesity groups. Multimorbidity prevalence was the highest among African Americans and lowest among Asian/Pacific Islanders. Even when factoring in age and weight class, the differences remain. The disease composition of multimorbidity also varied by race/ethnicity. African Americans presented with the most distinct multimorbidities at an earlier age compared with other races/ethnicities. Asian/Pacific Islanders, Biracial, and Hispanic groups had no distinct multimorbidities among the middle-aged. By understanding how multimorbidity patterns may clinically present within specific patient populations, health care professionals can implement more structured care plans and provide more appropriate care. Traditional care plans focus on one chronic disease and do not consider the impact of multiple risk factors and multimorbidity. This "single-disease treatment" approach may be inadequate in patients with multimorbidity and result in the involvement of various specialists, as well as an increase in polypharmacy^43^. These findings can be used to inform public policy and to develop patient guidelines at various obesity levels and ages.

Our study observed differences in multimorbidity prevalence and composition between the middle-aged and elderly groups and between with and without obesity cohorts. Unique diseases and disease combinations were shared by all race/ethnic groups within the elderly cohorts that weren't shared by all in the middle-aged. For example, we observed that ICD-10-CM diagnosis codes M25: Other joint disorders, M54: Dorsalgia, I25: Heart disease, I12: Hypertensive chronic kidney disease, N18: Chronic kidney disease (CKD), I50: Heart failure, E87: Other disorders of fluid, electrolyte and acid–base balance, E03: Other hypothyroidism, and M79: Other and unspecified soft tissue disorders, not elsewhere classified, in the elderly group but not in the middle-aged group (Fig. 1c,d). In addition, these diseases were frequently paired with either I10: Hypertension or E78: Lipidemia, which is consistent with findings from other studies^44^. Interestingly, in the elderly cohort with obesity, I25: Heart disease was combined with either E78: Lipidemia, I10: Hypertension, or E11: Diabetes, in three of five multimorbidity triads shared by all race/ethnicities (Fig. 1d). The triads are similar to those found in other studies. For example, Lim et al. 2018 similarly found that hyperlipidemia, hypertension, and diabetes were the most prevalent in dyad and triad disease combinations^44^. However, no other studies have looked for disease combinations of size 4. Therefore, our study is likely to have identified new triads and combinations of more than three diseases.

We found that African Americans presented with the highest number of multimorbidities at an earlier age than patients of other race/ethnicities, consistent with results observed in other studies^6^. African Americans are exposed to more traumatic experiences and stressors, such as discrimination and poverty, earlier on in life, which produces additional health risks and contributes to worse health outcomes in later life^45,46^. Although mental health disorders are not prevalent, the earlier emergence of multimorbidity could result from psychological distress at an earlier age^47^.

We identified a higher number of multimorbidity patterns distinct to African Americans, including many combinations of three or four diseases in the elderly with obesity. Previous studies have primarily identified multimorbidities consisting of up to three diseases. Our findings of multiple combinations of four diseases suggest that patients present with complex disease profiles that have likely never been studied before. The level of social vulnerability of particular groups should also be considered, as a significant correlation between social vulnerability and the total number of chronic conditions was previously demonstrated, with depression/anxiety, obesity, and cardiovascular diseases being the most related^47^. Further, these diseases were present in many patterns for various racial groups.

Another combination distinct to elderly, African Americans without obesity (Fig. 3c) was E87: Other disorders of fluid, electrolyte, and acid–base balance and I10: Hypertension. Alterations in acid–base transporters have been linked to hypertension^48^. Converging evidence indicates a pathogenic role of combined high sodium and low potassium levels in the development of hypertension and hypertension-associated cardiovascular complications^49^. Both these diagnoses serve as potential precursors of CKD, which is present at a higher prevalence in African Americans than other race/ethnic groups. As the kidneys play a vital role in regulating body fluids, electrolytes, and acid–base balance, CKD and end-stage renal disease (ESRD) predictably result in multiple complications, including hyperkalemia, metabolic acidosis, and hyperphosphatemia^49,50^. The multimorbidity combination consisting of E11: Diabetes and F17: Nicotine dependence was a unique combination to the elderly without obesity as well as the middle-aged with and without obesity Native Americans. According to the Centers for Disease Control and Prevention, American Indian/Alaska Native youth and adults have the highest prevalence of cigarette smoking in the US compared with other racial groups. Additionally, the risk of developing diabetes is 30–40% higher among smokers compared with nonsmokers^51^.

Caucasians had the most multimorbidity combinations in middle-aged with obesity as well as the elderly with and without obesity cohorts that include mental disorders, such as F41: Anxiety and F32: Major depressive disorder. Breslau et al. demonstrated that Hispanics and Non-Hispanic African Americans were at lower risk for common internalizing disorders: depression, generalized anxiety disorder, and social phobia compared to Caucasians^52^. F41: Anxiety presented with I10: Hypertension in the list of patterns distinct to Caucasians for the middle-aged and elderly with obesity cohorts. One study reported that having an anxiety disorder was associated with a fourfold increase in the risk of developing hypertension^53^. Major depressive disorder coupled with lipidemia was also distinct to Caucasians and was only present in the elderly with obesity. Studies have shown that anxiety and depression may be associated with increased cardiovascular risks and abdominal obesity. One study specifically noted that increased dyslipidemia and obesity risk in patients with severe anxiety disorders and depression might be partly explained by chronic low-grade inflammation and smoking^54^. Nicotine dependence was present in any disease combination among Caucasians. Comorbidities associated with mental health related disorders can present significant challenges leading to increased disability, longer hospital stays, and increased mortality^55^. Additionally, challenges related to managed care communication between provider and patient is more significant for patients with mental health diagnoses^56^.

The biracial group had no distinct multimorbidity combinations in the middle-aged cohort and few in the elderly. Of the multimorbidity combinations presented, hypertension was the most common morbidity present in combination with diseases in the clinical categories of the circulatory system or endocrine, nutritional and metabolic disorders. These findings are similar to previous work, which reported only one connection between disorders of circulatory system and disorders of endocrine, nutritional and metabolic diseases, and immunity amongst the biracial group^20^. However, our study is the first to identify multimorbidity prevalence within this group.

Our results demonstrate that Asian/Pacific Islander groups did not have any distinct multimorbidity combinations among the middle-aged and few among the elderly. This is consistent with other studies that find that combined Asian groups are typically healthier than non-Whites^57^.

Hispanics had fewer distinct patterns than Caucasians, Native Americans, and African Americans for each cohort except the middle-aged without obesity. Further, there were no multimorbidities distinct to Hispanics among these same cohorts. The comparable rate of multimorbidity patterns could be explained by the Hispanic Paradox, which posits that better health and mortality outcomes in Hispanics compared with non-Hispanics is due to healthier immigrants migrating into the country, while unhealthy people leave^58^. Therefore, despite Hispanics being of low socioeconomic status, studies observe Hispanics experience similar or better health outcomes than non-Hispanic Whites^59^. Our results could also reflect the underrepresentation of Hispanics within our dataset, as Hispanic is considered an ethnicity rather than race and was not counted twice.

The results of our sensitivity analysis suggests that differences in median BMI distributions by race/ethnicity are not driving our results. While differences within median BMI ranges were statistically significant but typically different by only 1 or 3 points. Differences in multimorbidity patterns and prevalence may be attributed to other factors, such as social determinants of health (SDoH), as socio-cultural and behavioral factors have been shown to influence obesity disparities across races^60^. It is suggested that there is a bidirectional relationship between multimorbidity severity and sociodemographic and lifestyle risk factors^61^.

We observed that African Americans and Native Americans appeared to share a higher prevalence of certain disease combinations than other races/ethnicities. For example, in the subset of patterns shared by only some of the race/ethnic groups in the middle-aged with obesity. African Americans and Native American groups had similar prevalence for the following combinations: E11: Diabetes + M25: Other joint disorders, E11: Diabetes + M54: Dorsalgia, and M25: Other joint disorders + M54: Dorsalgia as displayed in our results for combinations shared by only some groups as shown in Fig. 2b. Further, in the middle-aged without obesity cohort, these two groups shared three patterns with diagnoses in the musculoskeletal disorders (those starting with M) and shared seven in the middle-aged with obesity cohort. The presence of dorsalgia in combination with hypertension and diabetes across these races may be attributed to a deficiency of Vitamin D. One study observed that vitamin D deficiency was common amongst African Americans and Hispanics and may contribute to cardiovascular disease and diabetes^62,63^ and can also be a source of leg pain, widespread pain, arthralgia, rib pain, and back pain^64^. Musculoskeletal disorders are also commonly seen in patients with Type 1 and Type 2 diabetes^65^. These findings suggest that these specific groups have particular needs and certain morbidities that contribute to the emergence of unique multimorbidities, further emphasizing the necessity of understanding multimorbidities by race/ethnicity.

Guidance on how best to manage the health needs of specific patients based on their multimorbidity profile could be beneficial both for patients and the providers providing care. Our findings suggest that multimorbidity profiles should also include information on race/ethnicity and obesity. For example, African Americans were more at risk for multimorbidity earlier, even when without obesity. This suggests that providers should be vigilant about screening for diseases within this group at earlier ages, regardless of obesity category, compared with other races/ethnicities. This research could further inform larger public health goals by directing initiatives towards what is most prevalent within the patient population and promoting health system design improvement through the provision of patient- and family-centered care approaches^66^. Healthcare costs and utilization are also significant implications for patients with multimorbidity. By understanding multimorbidity, identifying the patients who are most likely to be affected by it, and examining common disease patterns, we could identify patients who are most likely to incur healthcare costs^67^, thus potentially reducing the economic burden of care that would be incurred after multimorbidity develops.

This study only analyzed patterns that were frequent above the threshold of 5%. There are multimorbidity patterns frequent below that threshold. Many of those combinations could become more prevalent over time. As part of our future work, we plan to analyze disease patterns across time to better understand the complexity of multimorbidity across race/ethnicity.

This study has a few limitations. First, the study sample is limited to patients seeking healthcare during the study period. Therefore, our results may underrepresent diseases that are prevalent among groups that are less likely to seek healthcare. Second, although we can account for certain factors, the cross-sectional design of our study limits our ability to fully understand age and obesity as risk factors for the development of multimorbidity. These results suggest there are differences in multimorbidity prevalence and composition between the middle-aged and elderly groups, and between groups with and without obesity. However, due to the study design, we could not assess the progression of multimorbidities in these subgroups over time. Our study did not account for gender due to the complexity of adding an additional stratification. So, we could not understand the impact of gender on multimorbidity. Lastly, the collection and identification of race and ethnicity pose another limitation. The race and ethnicity data is driven by the Uniform Hospital Discharge Data Set (UHDDS) definitions and required reporting for hospitals, and thus may not reflect the accurate representation of these categories^68^. The classification of Hispanic ethnicity as a race in this dataset is a further limitation as those patients could be Native American, Black, White, Asian, or a mix of these. However, despite these limitations, our study is unique in that it included biracial and Native American groups that are typically not captured or analyzed alone. Additionally, the study population represents a national sample drawn from across the country and represents the patient population that physicians typically treat in the clinical setting. Although some groups were seemingly under-represented in the study population, we captured trends specific to each group by analyzing each racial group separately.

## Conclusion

To our knowledge, this is the first study to identify *specific* multimorbidity patterns by race/ethnicity, stratifying by age and obesity. This is the first study to identify the prevalence of multimorbidity diseases across these cohorts. We found that multimorbidity was more prevalent in African Americans regardless of age or obesity status, and multimorbidity emerges at an earlier age within this group. Our findings also demonstrate that there are multimorbidity combinations unique to racial/ethnic groups, particularly amongst the middle-aged cohort with or without obesity. Identifying the most common comorbidity clusters among specific patient populations by weight class provides important clues regarding the underlying mechanisms leading to disease co-occurrence in patients by race/ethnicity and age. This research supports the development of patient-centered care approaches by stratifying patients with obesity-associated multimorbidity and providing care specific to their unique clinical needs.

## Supplementary Information

Supplementary Information.
