# Supplementary material for: Multimorbidity patterns across race/ethnicity as stratified by age and obesity
Source: Sci Rep. 2022 Jun 11;12:9716. doi: 10.1038/s41598-022-13733-w (PMC9188579; doi:10.1038/s41598-022-13733-w)
Supplement: Supplementary file 1 — Supplementary Information. [file 41598_2022_13733_MOESM1_ESM.docx]

**Supplementary Material**

**Supplementary Table 1. Patient Population.**

**Supplementary Table 2. ICD-10-CM Diagnoses included in the study.**

| **#** | **Diagnosis** |
| --- | --- |
| 1 | **D63:** Anemia in chronic diseases classified elsewhere |
| 2 | **D64:** Other anemias |
| 3 | **E03:** Other hypothyroidism |
| 4 | **E11:** Diabetes |
| 5 | **E55:** Vitamin D deficiency |
| 6 | **E78:** Lipidemia |
| 7 | **E86:**Volume depletion |
| 8 | **E87:** Other disorders of fluid, electrolyte and acid-base balance |
| 9 | **F03:** Unspecified dementia |
| 10 | **F10:** Alcohol related disorders |
| 11 | **F17:** Nicotine dependence |
| 12 | **F32:** Major depressive disorder, single episode |
| 13 | **F41:** Anxiety Disorders |
| 14 | **G47:** Sleep disorders are represented |
| 15 | **G89:** Pain, not elsewhere classified |
| 16 | **I10:** Hypertension |
| 17 | **I12:** Hypertensive chronic kidney disease |
| 18 | **I25:** Heart disease |
| 19 | **I48:** Atrial fibrillation and flutter are prevalent |
| 20 | **I50:** Heart failure |
| 21 | **I65:** Occlusion and stenosis of precerebral arteries, not resulting in cerebral infarction |
| 22 | **I73:** Other peripheral vascular diseases |
| 23 | **J44:** Other chronic obstructive pulmonary disease |
| 24 | **J45:** Asthma |
| 25 | **K21:** GERD |
| 26 | **K57:** Diverticular disease of intestine |
| 27 | **K59:** Other functional intestinal disorders |
| 28 | **M17:** Osteoarthritis of knee |
| 29 | **M19:** Other and unspecified osteoarthritis |
| 30 | **M25:** Other Joint Disorders |
| 31 | **M54:** Dorsalgia |
| 32 | **M79:** Other and unspecified soft tissue disorders, not elsewhere classified |
| 33 | **M81:** Osteoporosis without current pathological fracture |
| 34 | **N17:** Acute kidney failure |
| 35 | **N18:** Chronic kidney disease (CKD) |
| 36 | **N28:** Other disorders of kidney and ureter, not elsewhere classified |
| 37 | **N39:** Other disorders of urinary system |
| 38 | **N40:** Benign prostatic hyperplasia |

**Supplementary Figure 1:** Confidence Interval Overlap for Elderly Patients with Obesity Shared by All Races & Average Prevalence <0.08.


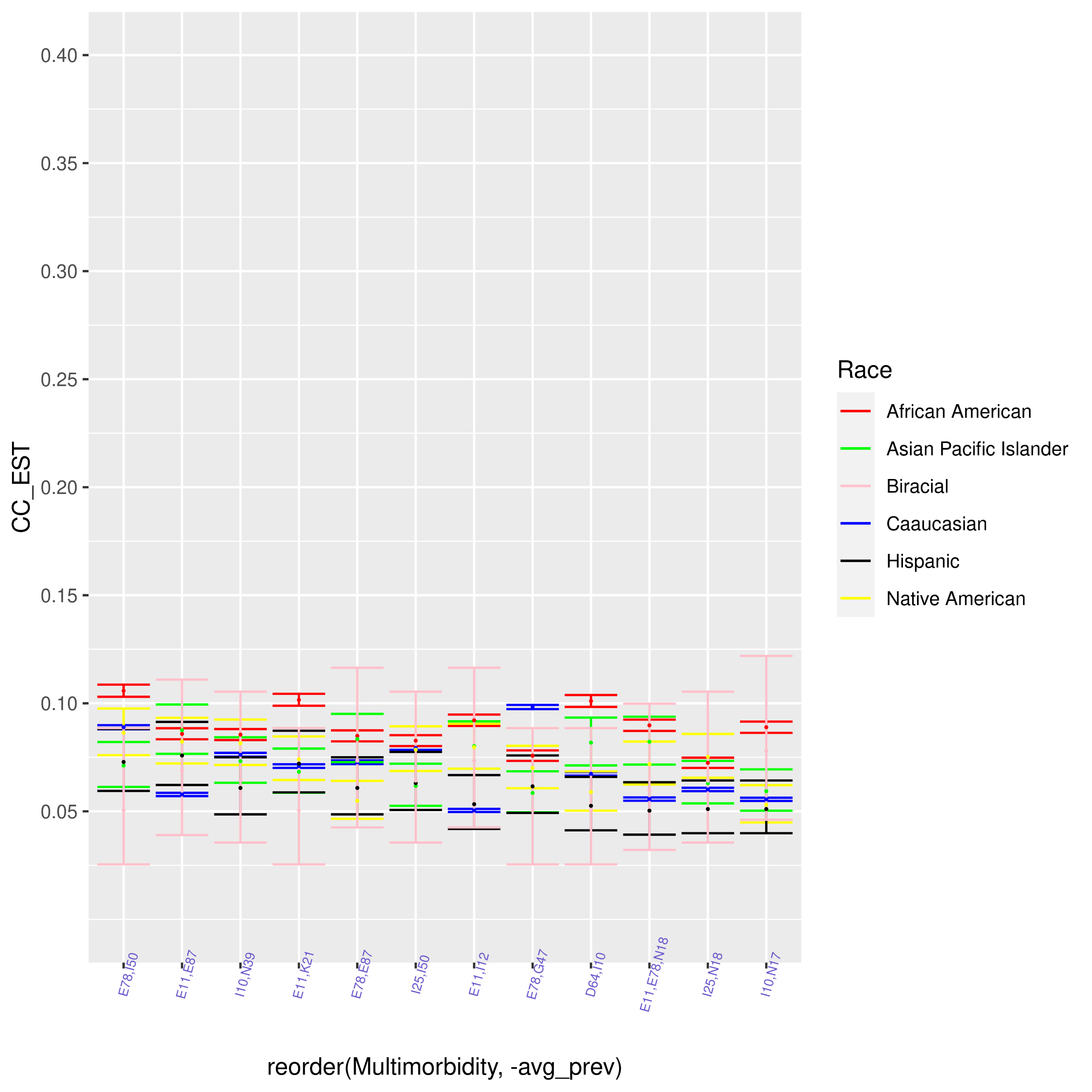


**Supplementary Figure 2:** Multimorbidities Shared by Some Races for Elderly Patients without Obesity & Average Prevalence < 0.065. Red box indicates non-significant g-test.


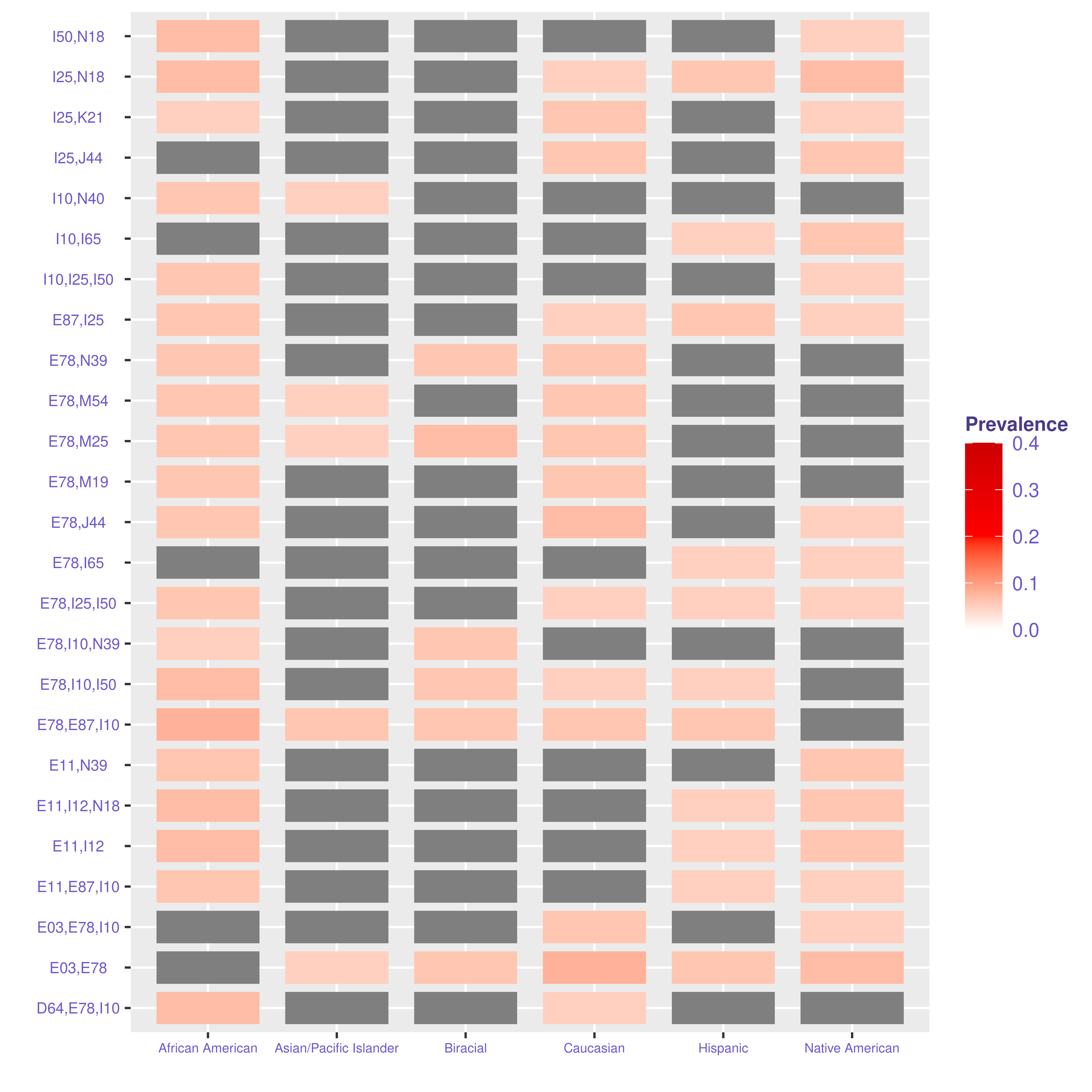

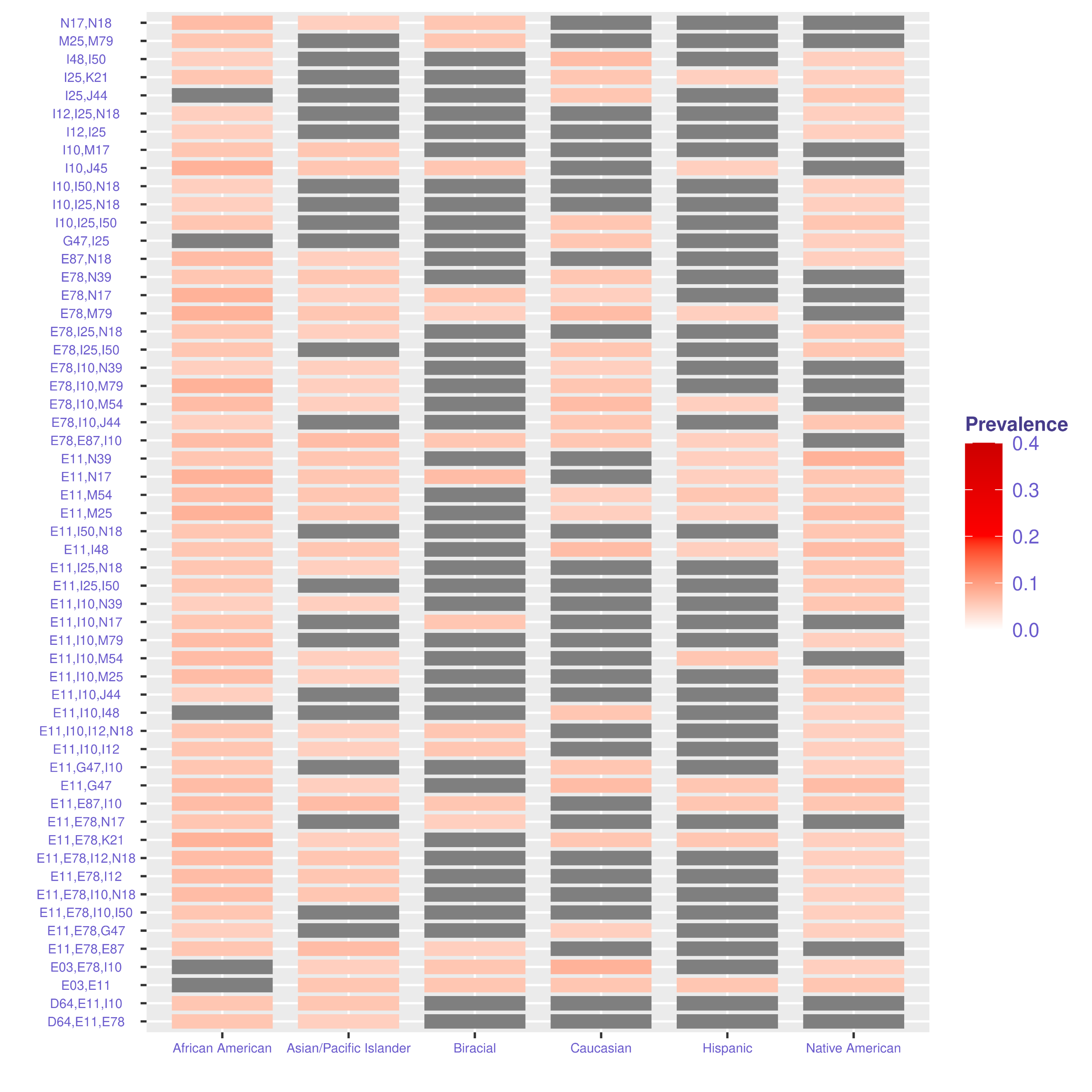


**Supplementary Figure 3:** Multimorbidities Shared by Some Races for Elderly Patients with Obesity ­& Average Prevalence <0.065.
